# Case report of *Salmonella* cross-contamination in a food laboratory

**DOI:** 10.1186/s13104-016-1969-7

**Published:** 2016-03-10

**Authors:** Geertrui Rasschaert, K. De Reu, M. Heyndrickx, L. Herman

**Affiliations:** Technology and Food Science Unit, Institute for Agricultural and Fisheries Research (ILVO), Brusselsesteenweg 370, 9090 Melle, Belgium; Faculty of Veterinary Medicine, Department of Pathology, Bacteriology and Poultry Diseases, Ghent University, Salisburylaan 133, 9820 Merelbeke, Belgium

**Keywords:** Salmonella, Cross-contamination, Food laboratory

## Abstract

**Background:**

This paper describes a case of *Salmonella* cross-contamination in a food laboratory. In 2012, chocolate bars shipped from Belgium to the USA were prevented from entering the USA because a *Salmonella* Rissen strain had been isolated from one of the chocolate bars in a Belgian food laboratory. However, a retrospective study of the *Salmonella* isolates sent from the laboratory to the Belgian National Reference Laboratory for *Salmonella* revealed that 7 weeks prior, a *Salmonella* Rissen strain has been isolated from fish meal in the same food laboratory. The chocolate bars were not expected to be contaminated with *Salmonella* because the ingredients all tested negative during the production process. Furthermore, because *Salmonella* Rissen is only rarely isolated from food, it was hypothesized that the two *Salmonella* Rissen isolates belonged to the same strain and that the second isolation event in this laboratory was caused by cross-contamination. To confirm this hypothesis, both *Salmonella* Rissen isolates were fingerprinted using different molecular techniques. To evaluate the discriminatory power of the techniques used, 11 other *Salmonella* Rissen isolates from different origins were included in the comparison. Pulsed-field gel electrophoresis, repetitive element palindromic PCR and three random amplified polymorphic DNA PCR assays were used.

**Results:**

Repetitive element palindromic PCR and random amplified polymorphic DNA PCR assays were insufficiently discriminatory, whereas pulsed-field gel electrophoresis using the combination of two restriction enzymes showed sufficient discrimination to confirm the hypothesis.

**Conclusions:**

Although cross-contamination in food laboratories are rarely reported, cross-contamination can always occur. Laboratories should therefore always be aware of the possibility of cross-contamination, especially when enrichment is used in the microbiological analysis. Furthermore, it is advised that results showing isolates of the same serotype isolated in a short time frame from unrelated food products should be interpreted carefully and should be confirmed with additional strain typing.

## Background

In April 2012, four containers of ready-to-eat chocolate bars were shipped from Belgium to the USA. Before they arrived in the USA, a sample of the chocolate tested positive for *Salmonella* in a Belgian accredited food laboratory using the ISO 6579 standard. The isolate was sent to the Belgian National Reference Laboratory for *Salmonella* (VAR, Veterinary and Agrochemical Research Centre), where it was serotyped as *Salmonella* Rissen. These results were communicated to the Belgian Federal Agency for the Safety of the Food Chain (FASC) and the US Food and Drug Administration (FDA). The FDA then blocked the containers at arrival in the USA, where 90 samples from the four containers of chocolate bars were taken for *Salmonella* detection and the container with the batch of presumptively *Salmonella* positive chocolate bars was destroyed.

However, the chocolate was not expected to be positive for *Salmonella* because the raw materials had tested negative during earlier steps in the production chain. Furthermore, *Salmonella* Rissen is a serotype that is known to be associated with primary production (e.g. feed products) but is found less frequently in foodstuffs [1]. *Salmonella* isolates from this laboratory were always sent to the National Reference Laboratory for *Salmonella* (VAR) for further serotyping. A retrospective study of all *Salmonella* isolates sent from the food lab revealed that in February 2012, 7 weeks before the chocolate bars were analyzed, *Salmonella* Rissen had been isolated from fish meal in the same food lab. That was the first time that a *Salmonella* Rissen strain had been isolated in that laboratory since September 2009. This led to the hypothesis that the batch of chocolate bars was in fact *Salmonella*-free and that cross-contamination in the food laboratory had led to a false-positive result, leading to severe economic consequences for the chocolate bar manufacturer.

## Results

**Fig. 1:**
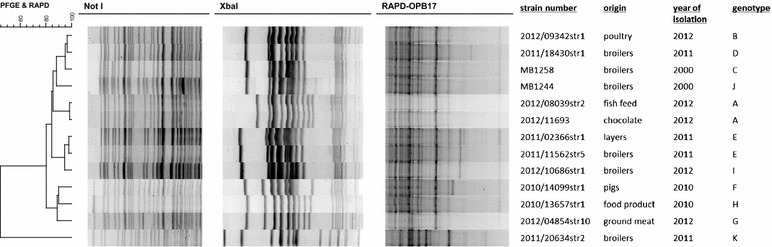
Cluster of the composite dataset of the fingerprints of 13 *Salmonella* Rissen isolates obtained by pulsed-field gel electrophoresis (restriction enzymes *Not*I and *Xba*I) and random amplified polymorphic DNA PCR (primer OPB-17). The similarities between the fingerprints were calculated using the Pearson correlation (optimization 1 %, tolerance 1 %) and the fingerprints were grouped using the UPGMA algorithm

Using primer (set)s ERIC and (GTG)_5_ for repetitive element palindromic PCR (rep-PCR) and primers 23L and P1254 for random amplified polymorphic DNA PCR (RAPD), the results were the same: only two fingerprints differed among the collection of 13 isolates. Each time, only one *Salmonella* Rissen isolate (2011/20634str2) had a different pattern from the other 12 isolates. With RAPD using primer OPB17, five different fingerprints were obtained. With pulsed-field gel electrophoresis (PFGE), the restriction enzymes *Xba*I and *Not*I each led to the distinction of ten pulsotypes (Fig. 1). After combining all the results, 11 genotypes were obtained.

Isolates 2011/02366str1 and 2011/11562str5 shared the same profile, as did the two isolates of interest 2012/08039str2 and 2012/11693. It was concluded that PFGE using the two restriction enzymes was sufficiently discriminatory and that the two *Salmonella* Rissen isolates detected in the routine laboratory were indeed the same strain. Because these results supported the hypothesis of cross-contamination, they were sent to the FDA and the remaining containers were released. Upon request of the FDA, the *Xba*I pulsotype of the chocolate isolate was also compared to the PulseNet USA database. Only one human *Salmonella* Rissen isolate had the same fingerprint, but this strain had been isolated prior to 2004 (pers. comm., Center for Disease Control).

## Discussion

In the Belgian laboratory, a *Salmonella* Rissen strain had been isolated from feed. Seven weeks later, a food product appeared to be contaminated with the same serotype, but it was hypothesized that the positive result was due to cross-contamination in the lab. The company, facing tremendous economic losses, wanted the fastest possible determination of whether the chocolate was *Salmonella* free, so the remaining containers could be released for delivery. Because cross-contamination was expected, the source of the presumptively-isolated *Salmonella* strain needed to be traced quickly. For a quick result, we used rep-PCR and RAPD but we also applied the more time-consuming PFGE because it is considered the gold standard for several pathogens including *Salmonella.* For the *Salmonella* Rissen serovar, the discriminatory power of these techniques was unknown as *Salmonella* serovars can vary greatly in genetic diversity [2–4]. Other *Salmonella* Rissen isolates were thus also included to evaluate the discriminatory power of the chosen techniques. Although the number of isolates was limited, it was concluded that PFGE—especially with the combination of both restriction enzymes- had sufficient discriminatory power to distinguish between different *Salmonella* Rissen isolates, whereas RAPD and rep-PCR were less suitable for discriminating between *Salmonella* Rissen strains.

It was concluded that the strain isolated from the fish meal was the same as the one presumptively isolated from the chocolate sample. Only sequencing techniques can yield absolute confidence that the isolates are 100 % identical, however. At the time of this writing, next-generation sequenced based techniques for sequencing whole genomes are now emerging and will be the techniques of the future for genotyping purposes, but these were not yet routinely in use at the time of the cross-contamination event (2012).

Although we are quite sure that the presumptive isolation of *Salmonella* Rissen from chocolate is due to cross-contamination, we still were faced with the question, “How could cross-contamination have occurred in this food laboratory?” The possible risk of cross-contamination when working with DNA amplifying techniques is well-known [5] . In literature, many cases of this type of cross-contamination can be found, e.g., false-positive cases of *Mycobacterium tuberculosis* or *Borrelia burgdorferi* (Lyme disease) in clinical settings [6]. In contrast, reports of cross-contamination using culture-based techniques in the laboratory are rather scarce, with the exception of misdiagnosing patients with *Mycobacterium tuberculosis.* It has been estimated that approximately 3 % of the reported *Mycobacterium tuberculosis* cases are false-positive due to cross-contamination in the laboratory [6, 7]. This high rate is due to the resistant nature of this bacterium, the ability to be transmitted via aerosols and batch-processing of multiple specimens [6, 7]. Similar to food products, the number of target bacteria is often low and many analyses include enrichment steps with the aim of multiplying the few target bacteria to detectable levels. For *Salmonella* isolation according to ISO 6579, the pre-enrichment step consists of using Buffered Peptone Water to multiply a very small number of *Salmonella* cells up to levels of 10^5^–10^7^colony forming units per ml after enrichment (depending of the food type and consequently the competitive flora). The high bacterial load after enrichment for *Salmonella* theoretically results in a high chance for cross-contamination. During a retrospective study from 2000 to 2007, 23 incidents of possible *Salmonella* cross-contamination had been identified in Irish labs [8], most of them food laboratories. Identified sources of cross-contamination were strains used as positive control, strains isolated previously from other food samples and strains used in proficiency test samples [8, 9]. For use as a positive control, the general recommendation is to use a less-common *Salmonella* serovar. In the above-described case, *Salmonella* Choleraesuis was used as positive control strain. The most probable causes for cross-contamination were new staff, clerical errors such as mislabeling, the use of insufficiently disinfected automated pipettes and the use of such pipettes without filter tips [8, 9].

In the case of the false-positive result for the chocolate bars, however, the 7-week time interval between the two isolation events makes the abovementioned explanations rather unlikely. In this particular case, the cause of cross-contamination will probably never be entirely elucidated, but some other explanations seem plausible. First, fish meal is a very powdery substance. When a subsample was weighed to start the analysis, it is possible that some fish meal dust flew into the air in the laboratory, where it continued to circulate in the lab environment for several weeks. Second, the *Salmonella* Rissen strain isolated from fish meal was stored in a slant tube. It is therefore also possible that an employee handled the slant improperly, thus leading to cross-contamination of the laboratory equipment, working tables, doors, etc. For these reasons, the good laboratory practices as described in ISO7218 recommend having separate locations for all steps in microbiological handling (including a separate location to subsample powder samples and make primary and subsequent dilutions), to clean and disinfect potentially contaminated surfaces and to test the laboratory equipment and air on a regular basis. This case also stresses the importance of analyzing blank samples, although the low number of blank analyses performed alongside the multitude of routine food samples does not guarantee detection of cross-contamination.

Even when all of the above precautions are taken, cross-contamination can still occur. Personnel working in routine laboratories should always be aware of the possibility of cross-contamination, especially when enrichment is used in the microbiological analysis. Therefore, results should be interpreted carefully, especially when the same rare serotypes are isolated from non-related food items in a short period of time. In case of doubt, additional strain typing should be used to determine if cross-contamination is a probable cause of the second isolation event.

## Conclusion

Cross-contamination in a food laboratory led to a false-positive *Salmonella* result for exported chocolate bars. Despite the large number of cross-contamination events described in clinical laboratories, especially when working with DNA amplifying techniques, similar reports of cross-contamination in food laboratories are rather scarce. The likely explanation is that cross-contamination in food is less likely to be detected. Because the target pathogen can be present in very low levels in the food, many microbiological food analyses include enrichment steps to multiply the target pathogen to detectable levels. Consequently, even a very low level of contamination in the lab environment can cause false-positive results when testing foodstuffs for microbiological food safety. Due caution is required when interpreting positive results, especially when the same species or serotypes are isolated from non-related food items in a short time span. In case of doubt, additional strain typing should be performed.

## Methods

To investigate the cross-contamination hypothesis, the two *Salmonella* Rissen isolates (from the chocolate bars and the fish meal) were fingerprinted using various molecular techniques. To evaluate the discriminatory power of the techniques used, 11 other *Salmonella* Rissen isolates from different origin were included in the tests (Fig. 1). These *Salmonella* isolates were all isolated in Belgium from 2000 to 2012 and were all from animal origin (pigs, broilers, layers) or food origin (e.g. ground meat). These isolates were kindly provided by VAR or from our own ILVO bacterial culture collection.

PFGE was performed according to the PulseNet protocol [10], with *Xba*I as the primary enzyme of choice (running conditions 19 h, 2.16–63.8 s) and *Not*I as secondary enzyme (24 h, 2–10 s). This technique takes 4–5 days to perform. In parallel, faster molecular techniques were performed. First, rep-PCR with the ERIC primer set or with the (GTG)_5_ primer was used. In most cases this technique discriminates at or just below the *Salmonella* serotype level [3]. Second, three RAPD PCR assays with the primers 23L, OPB17 and P1254 were evaluated [4]. Both rep-PCR and RAPD were performed on bacterial cell lysates made in 0.05 M NaOH—0.125 % (wt/vol) sodium dodecyl sulfate (SDS) and heated at 90 °C for 17 min. All gels were stained with ethidium bromide and digitally captured under UV light. The gel images were analyzed using BioNumerics 6.5 (Applied Maths, Sint-Martens-Latem, Belgium). If two isolates differed from each other by one band with any of the techniques performed, the two isolates were considered to be different strains.
